# Maternal mental health impacts 2-year cognitive and language outcomes in infants born before 30 weeks of gestation

**DOI:** 10.3389/fped.2026.1942213

**Published:** 2026-09-30

**Authors:** Danielle Clifford, Deirdre M. Murray, Brian H. Walsh

**Affiliations:** 1 Infant Centre, University College Cork, Cork, Ireland; 2 Department of Paediatrics and Child Health, University College Cork, Cork, Ireland; 3 Department of Paediatrics, Cork University Hospital, Cork, Ireland; 4 Department of Neonatology, Cork University Maternity Hospital, Cork, Ireland

**Keywords:** cognitive, environmental, mental health, outcome, premature

## Abstract

**Introduction:**

Parental mental health (MH) plays a key role in stimulating development and is potentially amenable to intervention. Our aim was to determine whether maternal MH affects 2-year outcomes in infants born before 30 weeks of gestation.

**Methods:**

MH variables routinely available at booking were collected for infants born before 30 weeks of gestation in a tertiary neonatal unit. An abnormal outcome was defined as a Bayley score of ≤85 in the cognitive or language domains at 24 months of corrected age. Logistic regression was performed, adjusting for demographic and clinical variables.

**Results:**

Of the 204 infants included, the mean gestational age was 26.7 weeks (SD 2.0). Anxiety was documented in 8.8% (18) of mothers, depression in 5.4% (11), anxiety/depression in 4.4% (9), and other MH diagnoses in 3.4% (7). Twelve mothers (5.9%) were prescribed MH medication at booking, and another 10 (5.0%) had required medication previously.

Infants whose mothers required medication had 3.8 (1.5–9.5) times the odds of a low cognitive and language score. After adjustment, this increased to 6.3 (1.6–25.5) for cognition but remained similar for language. The mean cognitive score was 88.9 (SD 19.9) for infants whose mothers required medication, compared to 99.0 (SD 17.6) for those who did not (*p* = 0.03). The mean language score was 85.4 (SD 20.1) for infants whose mothers required medication, compared to 98.5 (SD 18) for those who did not. Infants whose mothers required medication were more than twice as likely to have low cognitive and language scores (45% vs. 21% and 50% vs. 24%, respectively).

**Discussion:**

Maternal MH, at a severity that requires pharmacological treatment, may increase the odds of low cognitive and language scores in infants born before 30 weeks of gestation. Infants whose mothers required medication were more than twice as likely to have low scores. These families may benefit from additional support.

## 1 Introduction

Prediction of outcome in infants born extremely preterm remains difficult (1). As the focus shifts from broad predictions of composite outcomes of death or neurodevelopmental impairment towards more specific outcome measures, the search for prognostic indicators expands (2–6). While strides have been made in recent decades in the prediction and early detection of motor outcomes such as cerebral palsy, predicting cognition remains more challenging. It has been shown that social and environmental influences, such as maternal education and a cognitively stimulating home environment, play an important role in cognitive development (3). Aspects of an infant's environment that are potentially amenable to intervention are of specific interest, such as maternal mental health. Preterm birth has been highlighted as an important factor that may affect parental mental health, with higher rates of psychological distress reported, especially among those whose infants were at higher medical risk (7, 8). However, evidence suggests that there is no significant difference in rates of mental health disorders among mothers who delivered very preterm compared to those who delivered full-term, either 5 years after birth or over their lifetime (9). This highlights the importance of differentiating psychological symptoms from mental health diagnoses and investigating their influence on outcome separately to determine which, if either, plays a role in the development of high-risk infants. Therefore, in this report, we focus on maternal mental health diagnoses that exist prior to delivery.

Parental mental health is known to impact childhood outcomes in the general population, with the Millennium cohort in England reporting an association between high parental psychological distress and lower cognitive outcomes at school entry (10). Depressive symptoms and a diagnosis of maternal depressive and anxiety disorders have been most frequently reported to be associated with cognitive outcomes (11–13). There are limited data regarding the impact of other parental mental health diagnoses. A small study investigating infants of mothers at risk of post-partum psychosis (defined as a diagnosis of bipolar disorder, schizoaffective disorder, or a previous history of post-partum psychosis, *n* = 43) reported adverse cognitive outcomes at 12 months, assessed using the Bayley Scales of Infant and Toddler Development (BSID) III, compared to healthy controls (14). This difference remained significant after controlling for maternal full-scale intelligence quotient. Parental mental health may affect their interaction with their child and, therefore, their ability to provide a stimulating environment to support their development. Evidence on the role of the cognitively stimulating home environment and its influence on later cognitive outcome is established in the general population and is growing in high-risk infants, with suggestions of a critical role for those infants with brain injuries in particular (15–17). To date, there is little evidence on the influence of parental mental health on cognitive outcomes of very or extremely preterm infants. A Canadian study of 88 very low birth weight (VLBW) children from the nineties showed that early maternal anxiety traits in the NICU predicted cognitive and behavioural outcomes at 2 years corrected age (18). However, in an alternate Australian cohort of 143 infants born before 30 weeks of gestation, very little evidence was found that self-reported postnatal depressive or anxiety symptoms in the first month of life were associated with cognitive ability at 4–5 years (19). These findings were echoed in a US-based VLBW cohort of approximately 800 infants, where no significant difference was found in BSID scores between children of mothers with and without depressive symptoms at baseline or at preschool age (20). Interestingly, in this cohort, mothers with depressive symptoms were shown to perceive social abilities of their children more negatively than their teachers, which was not found to be the case for either those mothers without depressive symptoms or for infants who were born at term (20). Preterm infants whose mothers experienced depressive symptoms were also approximately one-third less likely to participate in preschool-age activities such as sports, dance, music, creative arts, or arts and crafts, which was not the case for the term-born cohort (20). These activities may provide important age-appropriate cognitive stimulation that can aid in school-readiness development and highlight the potential impact of parental mental health on the cognitively stimulating environment. Of note, none of these studies investigated the effects of a maternal mental health diagnosis on cognitive outcomes, but rather reported on depressive or anxiety symptoms or psychological distress generally. There is also a gap in the literature as to whether it is the type of mental health disorder, the severity, the timing or the chronicity that is most important.

This study aimed to determine whether maternal mental health, prior to delivery, is associated with 2-year cognitive and language outcomes in infants born before 30 weeks of gestation and to investigate which factor is most important in identifying families at highest risk.

## 2 Materials and methods

This was a retrospective single-centre study. Ethical approval was obtained from the Clinical Research Ethics Committee of the Cork Teaching Hospitals [ECM 4 (c) 30/07/2024 and ECM 3 (aa) 25/11/2025]. Infants born before 30 + 0 weeks of gestation at a single tertiary neonatal intensive care unit in Ireland between 2017 and 2022 were eligible. In addition to gestational age, inclusion criteria included the availability of relevant maternal information and infant outcome data. We excluded infants who did not survive to 2 years corrected age, those whose mothers died around the time of or after delivery, and those who had a genetic diagnosis known to affect neurodevelopment.

Infants were identified through the Vermont-Oxford Network database, and relevant medical variables were extracted, including data relating to brain injury. Additional data were collected from electronic medical records for socio-demographic variables, outcome data, and all mental health variables, which are routinely collected at the antenatal booking visit. Antenatal booking visit data were extracted from the chart of the infant's biological mother who carried the fetus, hereafter referred to as the mother. Data collected included a self-reported diagnosis of a mental health disorder, categorized according to the SNOMED CT (Systematized Nomenclature of Medicine Clinical Terms) medical diagnostic coding system, which is integrated into the Maternal and Newborn Clinical Management System (the national perinatal electronic medical record system) on Cerner Millennium from Oracle Cerner (21). Information on current or previous medication for mental health and, where applicable, engagement with mental health services, as well as the need for inpatient mental health treatment, was also collected. There are no routinely collected postnatal mental health data in our electronic medical records, as community follow-up services do not use this system. The data therefore relate to pre-existing mental health conditions, not those that arise anew as a consequence of the preterm delivery.

Outcome was assessed according to the clinical standard of care between 24 and 30 months corrected age using the BSID. The BSID was administered by one of three trained assessors (a clinical psychologist, an occupational therapist, or a physiotherapist). The BSID III was used for the cohort of infants born between 2017 and 2021. Subsequently, there was a change in clinical practice, and those born in 2022 were assessed using the BSID IV. An abnormal outcome was defined as a composite score of ≤85 in the relevant domain at 24 months corrected age (i.e., one standard deviation below the mean). We investigated both cognitive and language outcomes due to the intrinsic links between cognition and early language development.

Statistical analyses were performed using R software, version 4.2.2 (R Foundation for Statistical Computing) (22–26). Descriptive statistics were used for demographic and baseline characteristics. Pearson’s chi-square, Spearman’s correlation, or Fisher's exact test was performed, as appropriate, for univariate analyses. A *p*-value of ≤0.05 was considered significant. Logistic regression was performed to investigate the relationship between a low cognitive or language score and clinical, demographic, and maternal mental health variables, initially using unadjusted models and subsequently adjusting for clinical and demographic variables significant in the univariate analysis.

Deprivation index was used as a reflective marker of the socio-economic status of the infant. This was based on the home address of the child, universally available in the medical record, using the Pobal HP Deprivation Index, an Irish government tool that profiles communities based on census data, accounting for 10 measures of the disadvantage level of an area, including educational attainment, employment status, and number of individuals living in the household (https://www.pobal.ie/pobal-hp-deprivation-index/). This index is normally distributed at a national level. The neonatal unit where this study was based is in a county that is considered marginally above the average socio-economic level, i.e., less deprived than the average.

## 3 Results

In this cohort of 204 infants, the mean gestational age was 26.7 weeks (standard deviation, SD, 2.0), the mean birth weight was 970 g (SD 307), and 99 (48.5%) were female (Table 1). An intracranial haemorrhage of any grade was present in 82 (40.2%) infants, and a severe brain injury (Grade 3–4 intraventricular haemorrhage or periventricular leukomalacia, PVL) was present in 21 (10%). Of the 204 infants, 43 had a cognitive score ≤85 (21%) and 49 had a language score ≤85 (24%). Fifty-nine infants (29%) had either a low cognitive or language score, or both. Gestational age and birth weight were significantly correlated with the 2-year outcome, as were Apgar scores at 1 and 5 minutes, assisted reproductive therapy, length of stay, steroid treatment for chronic lung disease, presence of a severe brain injury, and deprivation index (Table 2). Due to the high correlation between these variables, the subsequent logistic regression was adjusted for gestational age, Apgar score at 1 minute, assisted reproductive therapy, severe brain injury, and deprivation index only, as appropriate.

**Table 1:** Demographics.

Variable	Total *n* = 204
Gestational age, mean	26.7 weeks (SD^a^ 2)
Birth weight, mean	969.8 g (SD^a^ 307)
Sex	
Female	99 (48.5%)
Male	105 (51.5%)
Multiple (twin/triplet)	72 (35.3%)
Maternal age, mean	32.7 (SD^a^ 5.8)
Assisted reproductive therapy	
None	132 (76.7%)
Medical	1 (0.6%)
IVF	19 (11%)
Donor egg	4 (2.3%)
Donor sperm	3 (1.7%)
Donor embryo	1 (0.6%)
ICSI	2 (1.2%)
IUI	10 (5.8%)
Not documented	32 (15.7%)
Antenatal steroids received	196 (96.1)
Antenatal magnesium sulphate received	170 (83.3%)
Mode of delivery	
Vaginal	64 (31.4%)
Section	140 (68.6%)
Apgar at 1 min, mean	5.0 (SD^a^ 2.3)
Apgar at 5 min, mean	7.0 (SD^a^ 2.0)
Periventricular–intraventricular haemorrhage	
Grade 1	41 (20.1%)
Grade 2	22 (10.8%)
Grade 3	2 (1.0%)
Grade 4	17 (8.3%)
Periventricular leukomalacia	2 (1.0%)
Steroids for chronic lung disease	40 (19.6%)
Oxygen at 36 weeks	79 (38.7%)
Length of stay, mean	90.6 days (SD 36.9)
Deprivation index	
Very affluent	6 (2.9%)
Affluent	27 (13.2%)
Marginally above average	89 (43.6%)
Marginally below average	55 (27.0%)
Disadvantaged	22 (10.8%)
Very disadvantaged	5 (2.5%)
Extremely disadvantaged	0
Bayley results, mean (SD)	
Cognitive (*n* = 204)	97.9 (18.1)
Language (*n* = 202)	97.1 (18.7)
Motor (*n* = 202)	92.4 (14.9)
Social-emotional (*n* = 186)	104.3 (22.9)
Adaptive behaviour (*n* = 204)	90 (20.1)

SD, standard deviation.

**Table 2:** Univariate analyses of demographics and mental health variables with low Bayley scores.

Variable	Bayley cognitive ≤85 (*n* = 43)	Bayley cognitive >85 (*n* = 161)	*p*-value	Bayley language ≤85 (*n* = 49)	Bayley language >85 (*n* = 153)	*p*-value
Gestational age (weeks), mean	25.7 (SD^a^ 2.0)	27.0 (SD^a^ 1.9)	**<0** **.** **01** ^b^	25.8 (SD^a^ 2.1)	27.0 (SD^a^ 1.9)	**<0** **.** **01** ^c^
Birth weight (g), mean	864.7 (SD^a^ 246.4)	997.8 (SD^a^ 316.1)	**0** **.** **02** ^b^	863.4 (SD^a^ 264.1)	1,003.3 (SD^a^ 314.1)	**0** **.** **01** ^b^
Sex			0.18^c^			0.56^c^
Female	17 (39.5%)	82 (50.9%)		22 (44.9%)	76 (49.7%)	
Male	26 (60.5%)	79 (49.1%)		27 (55.1%)	77 (50.3%)	
Maternal age (years), mean	31.7 (SD^a^ 5.4)	33.0 (SD^a^ 5.9)	0.73^c^	32.7 (SD^a^ 5.9)	32.6 (SD^a^ 5.6)	0.28^c^
Assisted reproductive therapy	21 (48.8%)	51 (31.7%)	**0** **.** **04** ^c^	21 (42.9%)	50 (32.7%)	0.19^c^
Antenatal steroids received	41 (95.3%)	155 (96.3%)	0.68^d^	47 (95.9%)	147 (96.1%)	1.0^d^
Antenatal magnesium aulphate received	36 (83.7%)	134 (83.2%)	1.0^d^	38 (77.6%)	130 (85.0%)	0.22^d^
Mode of delivery, vaginal	12 (27.9%)	52 (32.3%)	0.58^c^	19 (38.8%)	44 (28.8%)	0.19^c^
Apgar score at 1 min, mean	4.0 (SD 2.0)	5.3 (SD 2.3)	**<0** **.** **01** ^b^	4.2 (SD^a^ 2.1)	5.3 (SD^a^ 2.4)	**0** **.** **01** ^b^
Apgar score at 5 min, mean	6.3 (SD 2.0)	7.2 (SD 1.9)	**<0** **.** **01** ^b^	6.4 (SD^a^ 2.0)	7.2 (SD^a^ 2.0)	**0** **.** **01** ^b^
Any intracranial haemorrhage	19 (44.2%)	63 (39.1%)	0.55^c^	19 (38.8%)	63 (41.2%)	0.77^c^
Severe brain injury	9 (20.9%)	12 (7.5%)	**0** **.** **02** ^d^	6 (12.2%)	15 (9.8%)	0.63^c^
Steroids for chronic lung disease	13 (30.2%)	27 (16.8%)	**0** **.** **02** ^d^	15 (30.6%)	25 (16.3%)	**0** **.** **01** ^d^
Oxygen at 36 weeks	19 (44.2%)	60 (37.3%)	0.63^d^	22 (44.9%)	56 (36.6%)	0.09^d^
Length of stay (days), mean	106.0 (SD 46.1)	85.2 (SD 32.1)	**0** **.** **01** ^b^	106.6 (SD^a^ 44.9)	84.1 (SD^a^ 31.5)	**<0** **.** **01** ^b^
Deprivation index			**0** **.** **05** ^c^			**0** **.** **01** ^d^
Mental health diagnosis (any, *n* = 45)	13 (30.2%)	32 (19.9%)	0.15^c^	15 (30.6%)	30 (19.6%)	0.12^d^
Anxiety (*n* = 18)	4 (9.3%)	14 (8.7%)	1.0^d^	6 (12.2%)	12 (7.8%)	0.39^d^
Depression (*n* = 11)	4 (9.3%)	7 (4.3%)	0.25^d^	4 (8.2%)	7 (4.6%)	0.47^d^
Anxiety/depression (*n* = 9)	4 (9.3%)	5 (3.1%)	0.10^d^	4 (8.2%)	5 (3.3%)	0.23^d^
Other (*n* = 7)	1 (2.3%)	6 (3.7%)	1.0^d^	1 (2.0%)	6 (3.9%)	1.0^d^
Mental health medications						
Prescribed at booking visit (*n* = 12)	6 (14%)	6 (3.7%)	**0.02** ^d^	6 (12.2%)	6 (3.9%)	0.07^d^
Prescribed at any point (*n* = 22)	10 (23.3%)	12 (7.5%)	**0.01** ^d^	11 (22.4%)	11 (7.2%)	**<0.01** ^c^
Mental health services						
Attending at booking visit (*n* = 7)	2 (4.7%)	5 (3.1%)	0.64^d^	3 (6.1%)	4 (2.6%)	0.36^d^
Attending in the past (*n* = 24)	7 (16.3%)	17 (10.6%)	0.30^c^	7 (14.3%)	17 (11.1%)	0.55^c^
Inpatient mental health treatment (*n* = 4)	1 (2.3%)	3 (1.9%)	1.0^d^	1 (2.0%)	3 (2.0%)	1.0^d^

a SD, standard deviation.

b Spearman’s correlation.

c Pearson’s chi-square.

d Fisher's exact test.

Statistically significant results are highlighted in bold.

With regard to mental health, 22% (*n* = 45) of mothers reported a diagnosis, with anxiety documented in 8.8% (*n* = 18), depression in 5.4% (*n* = 11), and a combination of anxiety/depression in 4.4% (*n* = 9). Other mental health diagnoses were reported in 3.4% (*n* = 7). Twelve (5.9%) mothers were prescribed medication for their mental health at the time of antenatal booking, and a further ten (5.0%) had required medication previously, all of whom had a diagnosis. Of those prescribed medication at booking, six were taking selective serotonin reuptake inhibitors, three were taking benzodiazepines, one was taking anti-psychotics, and two did not have the class of medication documented. Seven mothers (3.4%) reported attending mental health services at booking, and 24 (11.8%) had attended previously. Four mothers (1.9%) were documented to have required inpatient mental health treatment.

None of the reported mental health diagnoses were significantly associated with a low Bayley cognitive score in univariate analysis (Table 2). However, after adjusting for clinical and demographic variables, a diagnosis of combined anxiety/depression increased the odds of a low cognitive score, with an adjusted odds ratio (aOR) of 10.9 (95% confidence interval: 1.5–78.8; Table 3). Infants of mothers who required mental health medication had an aOR of 6.3 (95% CI: 1.6–25.5) for a low cognitive score at 2 years (Table 3). This was even higher in those actively prescribed medications at the booking visit [aOR 7.1 (95% CI: 1.4–37.6)]. Attending mental health services did not appear to significantly affect the outcome, nor did inpatient treatment, although these numbers were very small (*n* = 4), so we were underpowered to assess the effect. The mean cognitive score was 88.9 (SD 19.9) for infants whose mothers required medication for mental health, compared to 99.0 (SD 17.6) for those whose mothers did not [*p* = 0.03, Supplementary Figure 1(a)]. Infants of mothers who required mental health medication were more than twice as likely to have low cognitive scores than the overall cohort [10/22 (45%) vs. 43/204 (21%)].

**Table 3:** Odds ratios for low cognitive score (Bayley ≤85) at 2 years corrected age.

Mental health variable	Unadjusted odds ratio (confidence interval^a^)	Adjusted odds ratio^b^ (confidence interval^a^)
Mental health diagnosis		
Anxiety (*n* = 18)	1.2 (0.3–3.7)	1.0 (0.2–5.1)
Depression (*n* = 11)	2.5 (0.6–8.7)	1.2 (0.1–10.7)
Anxiety/depression (*n* = 9)	3.4 (0.8–13.8)	**10.9** **(1.5–78.8)**
Other (*n* = 7)	0.7 (0.0–4.4)	0.7 (0.0–5.0)
Mental health medications		
Prescribed at booking visit (*n* = 12)	**4.2** **(1.2–14.1)**	**7.1** **(1.4–37.6)**
Prescribed at booking or in the past (*n* = 22)	**3.8** **(1.5–9.5)**	**6.3** **(1.6–25.5)**
Mental health services		
Attending at booking visit (*n* = 7)	1.6 (0.2–8.0)	5.9 (0.6–56.7)
Attended in the past (*n* = 24)	1.7 (0.6–4.2)	1.2 (0.3–4.8)
Inpatient mental health treatment (*n* = 4)	1.3 (0.1–10.1)	NA

a 95% Confidence interval.

b Adjusted for gestational age, 1 min Apgar score, assisted reproductive therapy, severe brain injury, and socio-economic status.

Statistically significant results are highlighted in bold.

With respect to language outcome, again, no reported mental health diagnosis was significantly associated with a low BSID score, and logistic regression did not demonstrate significantly increased odds for any diagnosis in either the unadjusted or adjusted analyses (Tables 2, 4). Infants of mothers who required mental health medication had an aOR of 3.7 (95% CI: 1.3–10.5) for a low BSID language score at 2 years (Table 4). As before, attendance at mental health services did not appear to significantly affect the outcome, nor did inpatient treatment, although these numbers were small. The mean language score was 85.4 (SD 20.1) for infants whose mothers required mental health medication, compared to 98.5 (SD 18) for those who did not [*p* = 0.01, Supplementary Figure 1(b)]. Again, infants of mothers who required mental health medication were more than twice as likely to have low language scores in this cohort [11/22 (50%) vs. 49/202 (24%)].

**Table 4:** Odds ratios for low language score (Bayley ≤85) at 2 years corrected age.

Mental health variable	Unadjusted odds ratio (confidence interval^a^)	Adjusted odds ratio^b^ (confidence interval)
Mental health diagnosis		
Anxiety (*n* = 18)	1.7 (0.6–4.6)	1.8 (0.5–6.0)
Depression (*n* = 11)	1.9 (0.5–6.5)	1.6 (0.4–6.6)
Anxiety/depression (*n* = 9)	2.7 (0.6–10.5)	3.0 (0.5–15.4)
Other (*n* = 7)	0.5 (0.0–3.1)	0.6 (0.0–3.7)
Mental health medications		
Prescribed at booking visit (*n* = 12)	3.5 (1.0–11.6)	3.9 (0.9–15.7)
Prescribed at booking or in the past (*n* = 22)	**3.8** **(1.5–9.5)**	**3.7** **(1.3–10.5)**
Mental health services		
Attending at booking visit (*n* = 7)	2.5 (0.5–11.6)	2.9 (0.5–15.5)
Attended in the past (*n* = 24)	1.4 (0.5–3.4)	1.3 (0.4–3.9)
Inpatient mental health treatment (*n* = 4)	1.1 (0.1–8.5)	NA

a 95% Confidence interval.

b Adjusted for gestational age, 1 min Apgar score, and socio-economic status.

Statistically significant results are highlighted in bold.

Nineteen of those with any intracranial haemorrhage had a low cognitive score (23%). This was higher in those with a severe brain injury (Grade 3–4/PVL), where 43% (*n* = 9) had a low cognitive score. Regarding language outcome, 19 of those with any intracranial haemorrhage also had a low score, and six of those with a severe brain injury (29%) had an adverse outcome. Infants with a severe brain injury had 3.3 times (95% CI: 1.3–8.4) higher odds of a low cognitive score, which fell to 1.7 (0.3–8.6) after adjusting for gestational age, 1-minute Apgar score, assisted reproductive therapy, and deprivation index. For language, the odds were 1.3 (0.4–3.4), which fell to 0.7 (0.1–3.4) after adjustment. Of those with a severe brain injury, the mothers of five infants (23.8%) had a mental health diagnosis, one (4.8%) of whom required mental health medications. Three of these five infants had low cognitive and language scores. No significant association was found between mental health medication (either at booking or at any time point) and severe brain injury.

## 4 Discussion

We have shown that maternal mental health difficulties, at a severity that requires pharmacological management, increased the odds of low BSID cognitive and language scores at 2 years corrected age, in this cohort of infants born less than 30 weeks of gestation. This remained significant after correcting for gestational age, 1-minute Apgar score, assisted reproductive therapy, severe brain injury, and deprivation index. Infants of mothers who required medication for their mental health were more than twice as likely to have low cognitive and language scores at 2 years corrected age, with a difference of more than 10 points in mean BSID scores between the groups. However, regarding a mental health diagnosis, only a combined diagnosis of anxiety/depression was associated with low cognitive outcome. No other diagnosis was associated with either a low cognitive or a low language outcome.

Maternal mental health conditions were common in this cohort, with just over 1 in 5 mothers reporting a mental health diagnosis, most frequently anxiety. These rates are similar to those quoted for European populations, where 25.9% report a mental health disorder (27). This is lower than the rates of psychological distress symptoms quoted on screening of the general national population (approximately 40%), which may relate to our use of diagnosis rather than symptoms, as well as the fact that diagnoses were self-reported at the antenatal booking visit (28). Our cohort also tended to be from less deprived areas, which may be related to the unit being situated in a county that is marginally above average or due to socio-economic bias among those lost to follow-up. This is important to note, as higher rates of mental health problems have been documented in lower socio-economic groups in both general and high-risk populations (7, 28). Rates of diagnosis in our study were similar to those in an Australian cohort of very preterm-born children, where 17% of mothers had anxiety or depressive symptoms in a clinical range at 7 years (7). Of note, this study reported that mothers of children born very preterm displayed elevated psychological distress compared to mothers of full-term children, which was consistent through to adolescence. In addition, there was evidence that mothers of children at higher neonatal medical risk displayed increasing psychological distress over time (7). Therefore, the reported rates in our cohort could also have increased postnatally. The limited evidence to date in this population has reported on mental health symptoms rather than diagnosis or pharmacological management, with mixed results (18–20). The information used for our analysis is routinely available in the maternal medical chart and would be less resource-intensive to screen for those who may benefit from additional support than conducting additional assessments of psychological symptomatology.

Our findings show that the need for mental health medications at any time point is a significant predictor of cognitive and language outcomes. Given that this encompasses both those who were on medication historically and those actively prescribed medication during pregnancy, we speculate that the effect is indirect, potentially a result of the severity of the condition rather than a direct consequence of the medication itself. Furthermore, the prescribed medications were heterogeneous and act through a variety of pharmacological mechanisms. No significant association was found between mental health medication and brain injury, with a similar proportion of infants having an IVH among those whose mother did and did not require mental health medications. This is in keeping with evidence from the term-born population where siblings who were and were not exposed to antidepressants *in utero* have been shown to have a similar risk of neurodevelopmental disorders (hazard ratio 0.97) (29).

There is growing interest in social and environmental factors that may help identify infants at the highest risk of adverse outcomes following preterm delivery (3). Those that may be amenable to intervention are of particular importance, offering potential opportunities to optimize outcomes for these infants. Our findings may highlight a unique avenue to improve care for these families. There are several paths through which maternal mental health may affect neurodevelopment. Maternal mental health has been shown to influence parenting behaviours in preterm infants. Maternal depressive symptoms have been linked with lower sensitive and structured parenting behaviours, and maternal anxiety symptoms have been linked with higher sensitive and structured parenting behaviours in very preterm children, an association that was stronger in those with higher social risk (based on family structure, primary caregiver education, primary income earner occupation, language spoken at home, and maternal age at birth) (19, 30). There was also an association between parenting behaviours and cognitive ability, with less intrusive parenting significantly associated with higher IQ at 5 years (19, 30). Maternal mental health may also influence the parent–child interaction and thus the level of cognitive and language stimulation provided to the infant at home. Parental cognitive stimulation, both qualitative and quantitative, has been linked with cognitive development in preterm infants (16). Higher maternal scaffolding, which describes the informational content of verbal communication with toddlers, has been associated with improved outcomes in preterm-born children (17). Even after adjusting for social risk, the enriched home environment is associated with cognitive outcomes in preterm-born cohorts (15). Therefore, it is possible that efforts to improve maternal mental health may benefit infant development through multiple modalities.

This is, to our knowledge, the first investigation of the specific mental health factors influencing cognitive and language outcomes of very preterm infants. It is also one of the few studies looking at the clinical diagnosis, medications, and inpatient treatment aspects of mental health, which are routinely available in the medical charts, rather than psychological symptomatology, in high-risk infants, and could easily identify those in need of additional family support. Despite this and our large sample size, our study has some limitations. The number of mothers with a mental health diagnosis is low (*n* = 45), and approximately half of these have required medication (*n* = 22). As mentioned, the sociodemographic profile tended towards the more affluent. The relatively high mean BSID composite scores in this high-risk population are consistent with this. Some who did not have BSID results available may include those with more severe impairments who were unable to engage with the assessment. The retrospective nature of the study and the use of data routinely collected in clinical charts depend on the accuracy of those reporting and recording the information. Mental health history therefore was not confirmed as physician-diagnosed. Some children may be in foster or adoptive care since delivery, which would not necessarily be documented in the neonatal charts; therefore, the maternal mental health may not be a consistent influence in their home environment. Those who have different genetics from the mother, through the use of donor egg or donor embryo, were not analysed separately due to small numbers (*n* = 5); however, they may demonstrate a different pattern, given that any mental health influence would be purely environmental. This would be an interesting area for investigation if an appropriate sample size were achieved. Directions for future research include prospective studies in the area with larger sample sizes and investigation of postnatal maternal mental health, as well as the importance of paternal mental health in high-risk cohorts. Longer-term follow-up to assess for an increase in the effects noted here would also be beneficial, as demonstrated in other cohorts for maternal education. An interventional study to investigate this as a potential pathway to optimize outcomes for families would help to establish a robust evidence base.

Our findings may help clinicians pragmatically identify families who would benefit from additional postnatal supports to optimize both maternal mental health and infant neurodevelopment, which are intricately linked.

## Data Availability

The raw data supporting the conclusions of this article will be made available by the authors, without undue reservation.

## References

[1] Crilly CJ Haneuse S Litt JS. Predicting the outcomes of preterm neonates beyond the neonatal intensive care unit: what are we missing? Pediatr Res. (2021) 89(3):426–45. 10.1038/s41390-020-0968-5 32428926 PMC7276948

[2] Linsell L Malouf R Morris J Kurinczuk JJ Marlow N. Prognostic factors for poor cognitive development in children born very preterm or with very low birth weight. JAMA Pediatr. (2015) 169(12):1162. 10.1001/jamapediatrics.2015.2175 26457641 PMC5122448

[3] Clifford D Walsh BH Murray DM. The role of social determinants of health in the developing injured brain. Curr Opin Pediatr. (2025) 37:570–6. 10.1097/MOP.0000000000001505 40904252 PMC12594155

[4] Jansen L Van Steenis A Van Den Berg-Huysmans AA Wiggers-de Bruine ST Rijken M De Vries LS. Associations between neonatal magnetic resonance imaging and short- and long-term neurodevelopmental outcomes in a longitudinal cohort of very preterm children. J Pediatr. (2021) 234:46–53.e2. 10.1016/j.jpeds.2021.02.005 33577803

[5] Bowe AK Lightbody G Staines A Murray DM Norman M. Prediction of 2-year cognitive outcomes in very preterm infants using machine learning methods. JAMA Netw Open. (2023) 6(12):e2349111. 10.1001/jamanetworkopen.2023.49111 38147334 PMC10751596

[6] O'Meagher S Kemp N Norris K Anderson P Skilbeck C. Risk factors for executive function difficulties in preschool and early school-age preterm children. Acta Paediatr. (2017) 106(9):1468–73. 10.1111/apa.13915 28502114

[7] Yaari M Treyvaud K Lee KJ Doyle LW Anderson PJ. Preterm birth and maternal mental health: longitudinal trajectories and predictors. J Pediatr Psychol. (2019) 44(6):736–47. 10.1093/jpepsy/jsz019 30977828 PMC7967874

[8] Sandnes R Le Floch M Riquin E Nocus I Müller JB Bacro F. Parental stress and mental health outcomes following very preterm birth: a systematic review of recent findings. J Affect Disord. (2024) 355:513–25. 10.1016/j.jad.2024.03.154 38556094

[9] Yates R Anderson PJ Lee KJ Doyle LW Cheong JLY Pace CC. Maternal mental health disorders following very preterm birth at 5 years post-birth. J Pediatr Psychol. (2022) 47(3):327–36. 10.1093/jpepsy/jsab101 34664642

[10] Mensah FK Kiernan KE. Parents’ mental health and children’s cognitive and social development: families in England in the Millennium Cohort Study. Soc Psychiatry Psychiatr Epidemiol. (2010) 45(11):1023–35. 10.1007/s00127-009-0137-y 19823757

[11] Psychogiou L Russell G Owens M. Parents’ postnatal depressive symptoms and their children’s academic attainment at 16 years: pathways of risk transmission. Br J Psychol. (2020) 111(1):1–16. 10.1111/bjop.12378 30882908

[12] Koutra K Chatzi L Bagkeris M Vassilaki M Bitsios P Kogevinas M. Antenatal and postnatal maternal mental health as determinants of infant neurodevelopment at 18 months of age in a mother-child cohort (Rhea Study) in Crete, Greece. Soc Psychiatry Psychiatr Epidemiol. (2013) 48(8):1335–45. 10.1007/s00127-012-0636-0 23248031

[13] Comaskey B Roos NP Brownell M Enns MW Chateau D Ruth CA. Maternal depression and anxiety disorders (MDAD) and child development: a Manitoba population-based study. PLoS One. (2017) 12(5):e0177065. 10.1371/journal.pone.0177065 28542256 PMC5443487

[14] Biaggi A Hazelgrove K Waites F Bind RH Lawrence AJ Fuste M. Mother-infant interaction and infant development in women at risk of postpartum psychosis with and without a postpartum relapse. Psychol Med. (2024) 54(4):823–34. 10.1017/S0033291723002568 37706314

[15] Treyvaud K Inder TE Lee KJ Northam EA Doyle LW Anderson PJ. Can the home environment promote resilience for children born very preterm in the context of social and medical risk? J Exp Child Psychol. (2012) 112(3):326–37. 10.1016/j.jecp.2012.02.009 22480454

[16] Nelson PM Demir-Lira ÖE. Parental cognitive stimulation in preterm-born children’s neurocognitive functioning during the preschool years: a systematic review. Pediatr Res. (2023) 94(4):1284–96. 10.1038/s41390-023-02642-x 37231307 PMC10761195

[17] Smith KE Landry SH Swank PR. Does the content of mothers’ verbal stimulation explain differences in children’s development of verbal and nonverbal cognitive skills? J Sch Psychol. (2000) 38(1):27–49. 10.1016/S0022-4405(99)00035-7

[18] Zelkowitz P Na S Wang T Bardin C Papageorgiou A. Early maternal anxiety predicts cognitive and behavioural outcomes of VLBW children at 24 months corrected age. Acta Paediatr. (2011) 100(5):700–4. 10.1111/j.1651-2227.2010.02128.x 21214883

[19] McMahon GE Treyvaud K Spittle AJ Giallo R Lee KJ Cheong JL. Parental mental health and parenting behaviors following very preterm birth: associations in mothers and fathers and implications for child cognitive outcome. J Pediatr Psychol. (2023) 48(3):293–304. 10.1093/jpepsy/jsac094 36655518

[20] Silverstein M Feinberg E Young R Sauder S. Maternal depression, perceptions of children’s social aptitude and reported activity restriction among former very low birthweight infants. Arch Dis Child. (2010) 95(7):521–5. 10.1136/adc.2009.181735 20522473 PMC3158425

[21] Sheehan OM Greene RA McKernan J Murphy B Cahill C Cleary B. Introduction of a single electronic health record for maternity units in Ireland: outline of the experiences of the project management team. JMIR Form Res. (2023) 7:e38938. 10.2196/38938 37171841 PMC10221488

[22] Team RC. R: A Language and Environment for Statistical Computing. Vienna, Austria: R Foundation for Statistical Computing (2024).

[23] Sjoberg DD Whiting K Curry M Lavery JA Larmarange J. Reproducible summary tables with the gtsummary package. R J. (2021) 13:570–80. 10.32614/RJ-2021-053

[24] Wickham H Francois R Henry L Müller K Vaughan D. _dplyr: A Grammar of Data Manipulation_. R package version 1.1.4. (2023).

[25] Wickham H Vaughan D Girlich M. _tidyr: Tidy Messy Data_. R package version 1.3.1. (2024).

[26] Wickham H. Ggplot2: Elegant Graphics for Data Analysis. New York: Springer-Verlag (2016).

[27] Alonso J Lépine JP. Overview of key data from the European study of the epidemiology of mental disorders (ESEMeD). J Clin Psychiatry. (2007) 68(Suppl 2):3–9. PMID: 17288501. 17288501

[28] Hyland P Vallières F Shevlin M Bentall RP Butter S Hartman TK. State of Ireland’s mental health: findings from a nationally representative survey. Epidemiol Psychiatr Sci. (2022) 31:e47. 10.1017/S2045796022000312 35773999 PMC9281488

[29] Suarez EA Bateman BT Hernández-Díaz S Straub L Wisner KL Gray KJ. Association of antidepressant use during pregnancy with risk of neurodevelopmental disorders in children. JAMA Intern Med. (2022) 182(11):1149–60. 10.1001/jamainternmed.2022.4268 36190722 PMC9531086

[30] Gerstein ED Njoroge WFM Paul RA Smyser CD Rogers CE. Maternal depression and stress in the neonatal intensive care unit: associations with mother-child interactions at age 5 years. J Am Acad Child Adolesc Psychiatry. (2019) 58(3):350–8.e2. 10.1016/j.jaac.2018.08.016 30768416 PMC6414066

